# Development and Validation of a Food Frequency Questionnaire for Population of Adolescents in Croatia

**DOI:** 10.17113/ftb.59.01.21.6630

**Published:** 2021-03

**Authors:** Ana Močić Pavić, Sara Sila, Tena Niseteo, Iva Hojsak, Sanja Kolaček

**Affiliations:** 1 Children’s Hospital Zagreb, Klaićeva 16, 10000 Zagreb, Croatia; 2 University of Zagreb, School of Medicine, Šalata 2, 10000 Zagreb, Croatia; 3 J.J. Strossmayer University of Osijek, Faculty of medicine in Osijek, Josipa Huttlera 4, 31000 Osijek, Croatia

**Keywords:** food frequency questionnaire, 3-day food record, questionnaire validation, adolescents

## Abstract

**Research background:**

Food frequency questionnaire (FFQ) is an important method for the estimation of dietary intake in epidemiologic studies. The aim of the study is to develop a FFQ and evaluate its relative validity for adolescents 12 to 18 years old.

**Experimental approach:**

FFQ was developed from a previously validated youth/adolescent diet questionnaire (YAQ) by modifying it in order to include Croatian national foods. The final version of the FFQ (FFQ-m) comprised 87 food items. The reference method was a set of two 3-day food records (3DFR) administered twice during the 3 non-consecutive days, one month apart. The FFQ-m was administered approximately on the last day of the second applied dietary food record. Adolescents were recruited from randomly selected elementary and high schools in urban and rural areas of Croatia. FFQ-m was validated on a sample of 84 adolescents (70.2% female). Nutritional intake from FFQ-m and 3DFR were analysed for each participant. Spearman correlation coefficients (r) and Bland-Altman method were used to assess the validity of the FFQ-m compared to 3DFR. Anthropometric parameters were assessed in 78 adolescents.

**Results and conclusions:**

The mean nutrient intake estimated by the FFQ-m was higher than that of the 3DFR. The average correlation coefficient for energy and nutrients in our validation study was 0.40. On average, 76.5% of adolescents were classified in the same or adjacent quartile of the nutrient intake. Bland-Altman analysis showed good agreement with all macronutrients and some micronutrients (sodium, phosphorus, potassium, calcium, magnesium and iron). A simple self-administered questionnaire completed by adolescents is a valid tool for measuring energy and nutrient intake among adolescent population.

**Novelty and scientific contribution:**

This is the first FFQ developed and validated for population of adolescents in Croatia. It will contribute to further research of nutritional intake in the population of adolescents, especially those from the region.

## INTRODUCTION

Although the trends of adolescent overweight and obesity have levelled off in some European countries, the prevalence remains high (*1*). Therefore, nutritional intake and diet quality remains an important focus of scientific community. Furthermore, it is already well recognized that diet, along with environmental and life-style determinants, plays an important role in the aetiopathogenesis and, *vice versa*, in the prevention of several non-communicable diseases (*2*), particularly if it starts in early childhood (*3*). In order to understand the association between diet and different diseases, appropriate tools for dietary assessment need to be developed (*4*). Standard methods of assessment of dietary intake include 24-hour recalls, food diaries and food frequency questionnaires (FFQs) (*5*). Currently, all of the available methods for dietary intake assessment have their advantages and disadvantages (*5*). The choice, therefore, depends on the size of the studied population, price, objectives of the study, among others (*6*, *7*).

In clinical and epidemiological studies, the most commonly used dietary assessment tools are FFQs (*5*). Quantitative FFQ estimates frequency and quantity of consumption of different foods and beverages. Moreover, FFQ can assess consumption of food during the previous month or previous year. Their advantages over other dietary assessment tools are a lower respondent burden, cost effectiveness and time saving (*8*). They can also be used to estimate both micro- and macronutrient intake, as well as the intake of foods and/or food groups (*9*).

There has been limited research into the diet of Croatian adolescents, and therefore, development and validation of FFQ is necessary. FFQs are in most cases validated against other methods that assess dietary intake such as 3-day food records (3DFR) or 24-hour recalls (*5*). The aim of this study is, therefore, to develop a quantitative FFQ and evaluate its relative validity against two 3-day non-consecutive food records for adolescents living in Croatia.

## MATERIALS AND METHODS

### Study design

The study was conducted to evaluate the validity of the modified food frequency questionnaire (FFQ-m). A set of two 3-day food records (3DFR) was applied twice during 3 non-consecutive days (including one day of the weekend). The time interval between each dietary food record was approximately one month. The FFQ-m was administered in elementary or high school approximately on the last day of the second applied 3DFR (approximately one month after the first 3DFR).

FFQ-m and 3DFR were distributed and anthropometric parameters were assessed in randomly selected elementary and high schools in urban and rural areas in Croatia. Each participant was required to fill out an FFQ-m and two 3DFRs. Both 3DFRs were distributed in schools at the same time. Participants were instructed to fill out one 3DFR shorty after its distribution, and the second after approximately one month. A month after the distribution of the two 3DFRs, FFQ-m was administered on site (at school), so that the interviewer (a physician) was available to provide assistance and clarification of the questionable responses. The 3DFRs were self-administered by adolescents who were 13 years old or older. For children who were 12 years old, data were collected with the help of their parents. Participants or their parents were instructed to enter all consumed foods and drinks in the 3DFR during the 3 non-consecutive days (including one day of the weekend). They were instructed to use kitchen scales to measure the amounts, but when that was not possible, they were allowed to use household measures such as cups, tablespoons and teaspoons. All household measures were converted into grams by the dietitian.

### Participants

The study included healthy adolescents aged 12 to 18, recruited from randomly selected elementary and high schools in urban and rural areas in Croatia who responded positively to the invitation to participate in the study. Permission for the study was obtained from appropriate authorities, parents were informed about the survey by the school principals, and their written consent was obtained. Study was approved by Ethics Committee of the Children’s Hospital Zagreb (IRB number: 21102014).

Recruitment was carried out until a total of 100 included participants (71 girls and 29 boys) had not been reached. Exclusion criteria were chronic illness or family history positive for chronic intestinal diseases (celiac disease, inflammatory bowel disease, gastrointestinal carcinoma) and the use of elimination diets. Furthermore, participants who did not fill out correctly and provided all the questionnaires, or whose energy intake estimated by the FFQ-m was lower than 500 kcal (2092 kJ) or higher than 4000 kcal (16736 kJ) were excluded from the analysis. As proposed by Willet (*5*), self-reported intake of fewer than 500 kcal (2092 kJ) and greater than 3500 kcal (14644 kJ) per day by women and fewer than 800 kcal (3347.2 kJ) and greater than 4000 (16736 kJ) kcal per day by men are unlikely. Therefore, considering our cohort of adolescents whose energy needs are higher than those of adults, we used lower cut-off of 500 kcal (2092 kJ) and upper cut-off of 4000 kcal (16736 kJ) for both genders. Sixteen participants did not meet the inclusion criteria. Thus, 84 participants were included in the validation study.

### FFQ development and validation

FFQ was developed from previously validated youth/adolescent questionnaire (YAQ) (*10*), which is a quantitative FFQ. Aforementioned FFQ was modified in order to include Croatian national foods. The modified FFQ (FFQ-m) contained 87 different food items divided into 8 different food groups: (*i*) milk and milk products, (*ii*) cereals and grains, (*iii*) juices and sodas, (*iv*) fruits, (*v*) vegetables, (*vi*) snacks, (*vii*) meat, poultry, fish, eggs and fat, and (*viii*) fast food. Available frequencies of food consumption were: never, 1-3 times a month, once a week, 2-4 times a week, 5-6 times a week, once a day, 2-3 times a day, 4-5 times a day or 6+ times a day. Available portion sizes were small, medium and large, and participants were able to distinguish their usual portion size using three portion size photos (*11*).

The individual food record data obtained with the FFQ-m were analysed by Microsoft Office Excel 2007 worksheet that was generated using the food composition database Federal Food Key (Bundeslebensmittelschlüssel) v. 3.01 (BLS 3.01) database (*12*) and Fachmann-Kraut-Nährwerttabellen (FKN) database (*13*). For those foods that were missing from the databases, USDA (FoodData Central) (*14*) and/or Kaić-Rak and Antonić (*15*) databases were used, while for typical national Croatian foods, Kaić-Rak and Antonić (*15*) food composition database was used, or the nutritional composition was added from the nutritional label for Croatian brand products. The frequency of consumption of food items was multiplied by the portion size to calculate the amount of nutrients consumed in a 30-day period, from which an average daily energy and nutrient intake per each participant was determined. Intake of 24 nutrients was analysed: total protein, plant protein, total fat, saturated fatty acids (SFA), monounsaturated fatty acids (MUFA), polyunsaturated fatty acids (PUFA), cholesterol, total carbohydrates, mono- and disaccharides, polysaccharides, dietary fibre, sodium, potassium, calcium, magnesium, phosphorus, iron, zinc, retinol equivalent, vitamins B1 and B2, niacin, vitamin B6 and vitamin C.

Energy and nutrient intake estimated by the 3DFR was analysed using PRODI v. 5.7 software (*16*), which contains approx. 14 800 foods from the BLS v. 3.01 (*12*) and FKN (*13*), the same databases that were used for the analysis of FFQ-m. Commonly consumed Croatian foods were added into the database of the PRODI software using USDA (FoodData Central) (*14*) and Kaić-Rak *et al*. (*12*) food composition databases.

The preliminary results of this study have been presented previously (*17*). This manuscript adds the more thorough and rigid statistical analysis of the preliminary results and describes the results in more detail.

### Anthropometric parameters

Anthropometric parameters were assessed for 78 participants, since 6 participants did not show up at the time when anthropometric assessment was performed.

Anthropometric parameters, assessed for each participant, were: body mass (BM), body height (BH), body mass index (BMI) and BM-for-age, BH-for-age and BMI-for-age. BM was measured on an electronic scale (Seca, type 877; Vogel & Halke Gmbh & Co., Hamburg, Germany), with subjects wearing light-weight gym clothes. BH was measured with a portable stadiometer (Seca, type 217; Vogel & Halke Gmbh & Co., Hamburg, Germany). Nutritional status of participants was determined using World Health Organization (WHO) growth reference data for children and adolescents (5-19 years) (*18*).

### Statistical analysis

All consumed food items and meals estimated by the FFQ-m and 3DFRs were expressed as daily nutrient intakes, and correlation was used to identify their relationship. Normality of distribution was assessed using the Kolmogorov-Smirnov test. For most nutrients, the data were not normally distributed. Therefore, non-parametric methods (Spearman's correlation coefficients) were used to evaluate the validity of the FFQ-m against the two 3DFR. Comparisons of nutrient intakes estimated by the FFQ-m and 3DFR records were made using Wilcoxon signed-rank test. The energy and nutrient intakes estimated by the FFQ-m and 3DFR were categorized into quartiles (Q1=low, Q2=low to medium, Q3=medium to high, and Q4=high intake). The proportion of subjects categorized into the same, adjacent or opposite quartile was estimated. The average correlation coefficient for all nutrients, as well as macronutrients and micronutrients separately, was calculated as a sum of the correlation coefficient divided by the number of items (nutrients). Additionally, Bland-Altman analysis (*19*) using mean difference and 95% limits of agreement was conducted to graphically assess the presence of bias or disagreement. Two-tailed tests with level of significance α=0.05 were used for all statistical analyses. statistical software for the analysis was SPSS v. 19.0 (*20*).

## RESULTS AND DISCUSSION

This study shows that a simple self-administered questionnaire completed by adolescents is a valid tool for measuring energy and nutrient intake among adolescent population in Croatia. The mean nutrient intakes estimated by the FFQ-m were higher than those of the 3D-FR, while the average correlation coefficient for energy and nutrients in our validation study was 0.40. On average, 76.5% adolescents were classified in the same or adjacent quartile of the nutrient intake. In Croatia, very limited data on the diet of adolescents is available, therefore, this FFQ will make the assessment of the diet in a large population of adolescents simpler and less burdensome.

From 84 participants included into the final analysis, the main characteristics of 78 adolescents for whom anthropometric data were estimated are presented in Table 1. In total, 26.9% adolescents were overweight or obese and 14.1% of adolescents were underweight.

**Table 1:** Baseline characteristics of study population

Participant's characteristics	*N*_total_=78
Age/year	(15.35±1.87)
*N*_female_/%	(59±70.2)
BM-for-age	(0.13±1.11)
BH-for-age	(-0.08±0.99)
BMI-for-age	(0.23±1.07)

BM=body mass, BH=body height, BMI=body mass index, results are presented as mean value±standard error

The results of our FFQ-m validation study showed a good correlation with the 3DFR for the intake of all macronutrients and some micronutrients (sodium, phosphorus, potassium, calcium, magnesium and iron). To our knowledge, this is the first FFQ developed and validated for Croatian adolescent population. FFQs are low-cost, fast and easy to administer tools, and therefore commonly used in epidemiologic studies (*8*).

Average intakes of macro- and micronutrients estimated by the FFQ-m and 3DFR are presented in Table 2. A significantly higher intake of energy, most macronutrients (total protein, total fat, SFA, MUFA and PUFA, mono- and disaccharides and dietary fibre) and micronutrients (potassium, phosphorus, iron, retinol equivalents, vitamins B1, B2 and B6, niacin and vitamin C) were estimated by the FFQ-m and compared to the 3DFR. Overestimation of FFQ-m compared to 3DFR was 33.1% on average for macronutrients, ranging from 4.2% for total carbohydrates to 62.7% for PUFA. Overestimation of micronutrients ranged from 1.8% for calcium to 171.4% for vitamin B6. FFQ-m underestimated intakes of cholesterol, sodium, magnesium and zinc compared to 3DFR (Table 2).

**Table 2:** Absolute intake, differences in nutrient intake and correlation coefficients between FFQ-m and two 3DFRs

Nutrients and energy	3DFR *N*=84				FFQ-m *N*=84				Wilcoxon		Correlation coefficient	
Median		Range		Median		Range		z	
*E*/kcal	1604.8		763.6- 4918.8		1751.2		795.4-3294.45		-3.3*		0.70**	
*E*/kJ	6731.2		3194.9-20580.3		7327.0		3328.0-13784.0		-3.3*		0.70**	
*m*(total protein)/g	73.4		32.6-172.3		77.3		30.4-158.4		-2.5*		0.51**	
*m*(plant protein)/g	20.6		7.7-67.4		18.3		9.9-37.8		-1.5		0.40**	
*m*(total fat)/g	52.1		21.8-248.4		74.1		22.8-155.8		-5.8*		0.60**	
*m*(saturated fatty acids)/g	19.9		7.4-77.8		31.4		8.0-64.5		-6.2*		0.60**	
*m*(monounsaturated fatty acids)/g	15.6		6.6-51.7		25.2		6.9-67.0		-6.9*		0.63**	
*m*(polyunsaturated fatty acids)/g	7.5		2.4-84.0		12.2		4.7-26.6		-6.0*		0.45**	
*m*(cholesterol)/mg	212.2		71.5-611.4		188.3		13.1-672.3		-2.3*		0.50**	
*m*(total carbohydrates)/g	199.8		79.3-494.7		208.2		106.1-386.7		-1.6		0.56**	
*m*(mono- and disaccharides)/g	60.2		6.5-151.6		93.0		23.6-206.1		-6.8*		0.32**	
*m*(polysaccharides)/g	94.7	31.6-365.2		103.6		46.9-217.4		-1.5		0.50**	
*m*(dietary fibre)/g	14.7	6.3-30.6		18.3		7.6-41.7		-4.5*		0.30**	
*m*(sodium)/mg	1892.7	695.2-6188.7		1687.2		499.4-3890.6		-3.2*		0.49**	
*m*(potassium)/mg	2317.1	552.9-6747.8		2594.5		1384.9-5186.3		-5.2*		0.39**	
*m*(calcium)/mg	683.7	206.8-2168.9		696.2		257.5-1579.0		-1.5		0.37**	
*m*(magnesium)/mg	217.6	89.3-601.1		187.0		80.0-449.8		-3.0*		0.36**	
*m*(phosphorus)/mg	1018.1	339.5-2173.5		1253.5		630.0-2457.0		-5.2*		0.48**	
*m*(iron)/mg	9.0	3.8-23.9		10.5		5.0-20.0		-4.4*		0.47**	
*m*(zinc)/mg	8.9	4.7-19.0		6.7		3.2-11.5		-5.1*		0.16	
*m*(retinol equivalent)/IU	658.8	164.5-3816.8		998.2		314.3-5269.7		-4.1*		0.16	
*m*(vitamin B1)/mg	0.9	0.4-3.6		3.6		0.9-7.7		-7.9*		0.07	
*m*(vitamin B2)/mg	1.2	0.3-2.7		3.9		1.0-7.9		-8.0*		0.04	
*m*(niacin)/mg	24.1	8.7-65.8		34.8		9.6-169.4		-5.1*		0.23*	
*m*(vitamin B6)/mg	1.4	0.4-5.8		3.8		1.5-18.1		-8.0*		0.32**	
*m*(vitamin C)/mg	75.0			167.4		27.4-919.3		-7.6*		0.34**	
Average of correlation coefficients (energy and all nutritents)										0.40	
Average of correlation coefficients (energy and macronutrients)										0.51	
Average of correlation coefficients (micronutrients)										0.30	

3DFR=3-day food record, E=energy intake, FFQ-m=modified food frequency questionnaire

* and **difference is significant at p<0.05 (two-tailed) and p<0.01 (two tailed), respectively

The detected overestimation of energy and nutrient intakes has been observed in previous validation studies in children and adolescents as well (*21*-*26*). Our FFQ-m largely overestimated the intake of vitamins B1, B2 and B6, and therefore cannot be used for the assessment of these nutrients. Detected overestimation of FFQ in comparison with other dietary methods could be caused by a wide selection of available foods that the participant can choose from (*27*). Similarly, since 3DFR estimates nutritional intake on random days, consumption of some foods that are part of the FFQ-m might have been missed on days when the 3DFRs were filled. Indeed, frequent underreporting of food intake obtained from food records (*28*-*30*) and over-reporting in FFQs (*31*, *32*) have been observed in previous studies.

The correlation coefficients between FFQ-m and two 3DFRs are presented in Table 2. The Spearman’s correlation coefficient showed a significant relationship between the energy intake and all macro- and micronutrients except zinc, retinol equivalent and vitamins B1 and B2. The highest correlation between the two methods was shown for total energy (r=0.701), total fat (r=0.604), total protein (r=0.510), total carbohydrates (r=0.560), SFA (r=0.589) and MUFA (r=0.628).

The average correlation coefficient for energy intake and nutrients in our validation study was 0.40, which is in accordance with other studies (*22*-*33*). The highest correlation coefficient was detected for energy intake (r=0.7), similar to that of Morel *et al.* (*26*). When separated, in our study mean correlation for macronutrients (r=0.51) was higher than that of micronutrients (r=0.30), mostly due to low correlation coefficients for zinc, retinol equivalent, and vitamins B1 and B2. Similarly, in other studies correlation coefficients for macronutrients were higher than those of some micronutrients (*33*, *34*).

Cross-classification analysis (Table 3) revealed that on average, 76.5% adolescents were classified in the same or adjacent quartile of nutrient intake when comparing the data obtained by the 3DFR. On average, 5.1% adolescents were classified into the opposite quartile and 35.1% adolescents were classified into the same (identical) quartile.

**Table 3:** Number of participants ranked into the same, same and adjacent, or opposite quartiles of the distribution according to the nutrient estimates obtained from the FFQ-m and 3DFR (*N*=84)

Nutrient	*N*(participant)/%		
Same quartile	Same and adjacent quartile	Opposite quartile
*E*/kcal	50.0	86.9	1.2
*m*(total protein)/g	40.5	77.4	3.6
*m*(plant protein)/g	33.3	78.6	7.1
*m*(total fat)/g	41.7	84.5	0.0
*m*(saturated fatty acids)/g	42.9	82.1	3.6
*m*(monounsaturated fatty acids)/g	47.6	85.7	0.0
*m*(polyunsaturated fatty acids)/g	40.5	79.8	6.0
*m*(cholesterol)/mg	34.5	76.2	6.0
*m*(total carbohydrates)/g	34.5	75.0	2.4
*m*(mono- and disaccharides)/g	38.1	83.3	4.8
*m*(polysaccharides)/g	36.9	78.6	8.3
*m*(dietary fiber)/g	42.9	84.5	3.6
*m*(sodium)/mg	19.0	72.6	6.0
*m*(potassium)/mg	35.7	81.0	2.4
*m*(calcium)/mg	40.5	76.2	4.8
*m*(magnesium)/mg	28.6	75.0	3.6
*m*(phosphorus)/mg	38.1	75.0	6.0
*m*(iron)/mg	45.2	76.2	2.4
*m*(zinc)/mg	35.7	81.0	3.6
*m*(retinol equivalent)/IU	32.1	67.9	9.5
*m*(vitamin B1)/mg	23.8	66.7	7.1
*m*(vitamin B2)/mg	26.2	63.1	11.9
*m*(niacin)/mg	22.6	63.1	10.7
*m*(vitamin B6)/mg	25.0	63.1	2.4
*m*(vitamin C)/mg	34.5	79.8	7.1

3DFR=3-day for record, FFQ-m=modified food frequency questionnaire

These results are in accordance with the results of previous studies (*22*-*24*, *26*, *35*). The highest percentage of adolescents who were classified into the opposite quartile was observed for retinol equivalent, vitamin B1, vitamin B2 and niacin. The same nutrients have shown the lowest coefficient correlation, possibly for the same reasons that were discussed previously.

**Fig. 1:**
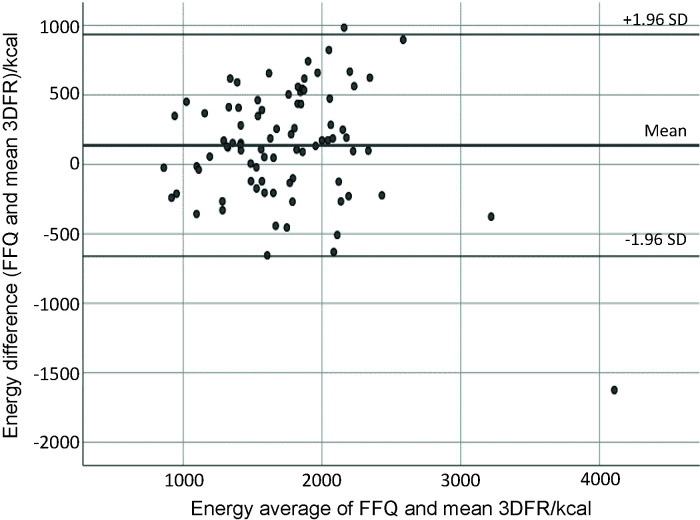
Bland-Altman plot showing agreement between the FFQ and 3D-FR for energy intake (kcal). FFQ=food frequency questionnaire, 3DFR=3-day food record

**Fig. 2:**
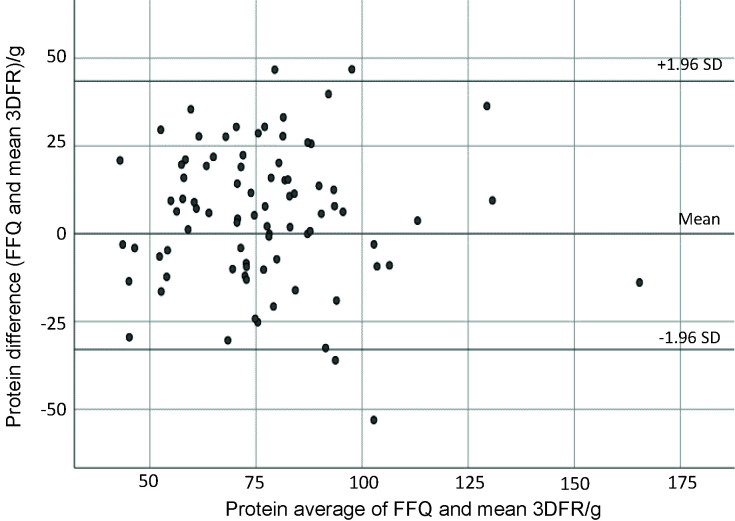
Bland-Altman plot showing the agreement between the FFQ and 3D-FR for protein intake (g). FFQ=food frequency questionnaire, 3DFR=3-day food record

**Fig. 3:**
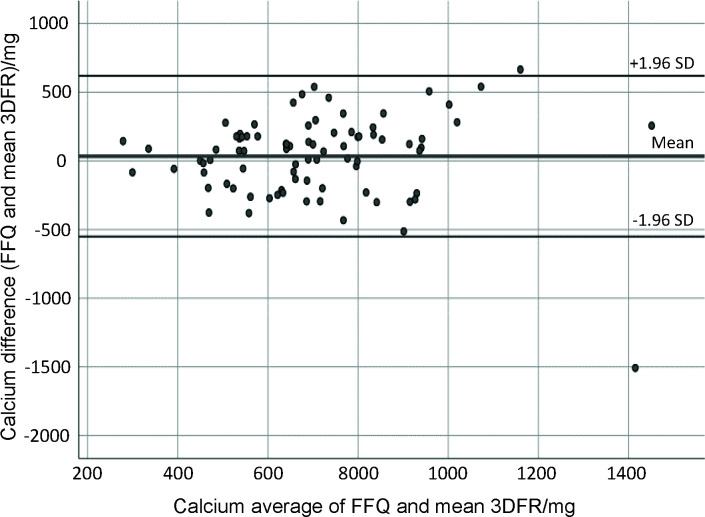
Bland-Altman plot showing the agreement between the FFQ and 3D-FR for calcium intake (mg). FFQ=food frequency questionnaire, 3DFR=3-day food record

Bland-Altman analysis is often used in validation studies in conjunction with correlation coefficients, and is suggested to assess absolute validity of the FFQ (*36*). This analysis assesses in graphical form the agreement between the methods across the range of intakes by plotting the mean of the two methods against the difference. The mean agreement indicates how well the FFQ and food records agree on average (*36*). The Bland and Altman plotting (*19*) showed good agreement among the total energy intake, total protein, total fat, SFA, MUFA, cholesterol, total carbohydrates, calcium, magnesium, potassium, phosphorus and iron. Figs. 1-3 present plots for energy, total protein and calcium intake, showing good agreement between the FFQ-m and 3DFR for all mentioned nutrients, where the Y axis shows the difference between the FFQ-m and 3DFR derived measurements and the X axis represents the average of these measures (*37*). Plots produced for other nutrients were similar to those shown in Figs. 1-3, except for retinol equivalent, vitamin B1, vitamin B2, niacin and vitamin B6, for which proportional bias was noticed (data not shown).

The main limitation of this study includes fairly low number of participants. Even though we were aiming to recruit the same number of male and female adolescents, male adolescents were more prone to fill out the questionnaires incorrectly and incompletely and were excluded from the study more often than the female adolescents. Additionally, female adolescents are more worried about their appearance and healthy eating habits, and therefore, more willing to participate in similar studies (*38*). Furthermore, one of the limitations of using 3DFR is that adolescents may change their diets, which creates a distortion of their usual intake (*39*, *40*). However, 3DFR is the best dietary method available for the assessment of dietary intake since it does not depend on the recall (*41*). Yet another limitation is that we were not able to use the national food database for the assessment of nutrient intake. Although Croatia has its own national food composition database (*15*), it is outdated and impractical to use. However, by combining different food databases, we were able to analyse all the meals and food items that are typical for the Croatian adolescent population. The last limitation is that FFQ-m was administered on only one occasion. By administering 3DFR and FFQ-m in two different seasons, we would have been able to capture difference in seasonal food intake.

There are also some strengths of this study. Most importantly, the comparison has been made to the 3DFR, which is considered to be the best available dietary method (*41*). Moreover, FFQ-m and two 3DFRs were applied for each participant within one month. This approach excludes all the possible dietary changes that would have occurred if a longer time period between the two 3DFRs had passed.

## CONCLUSIONS

In conclusion, the developed food frequency questionnaire (FFQ) and its validation study showed that the modified FFQ (FFQ-m) is a reliable tool to estimate the relative intake of energy, macronutrients and some micronutrients by adolescents in Croatia. It is affordable, quick and, most importantly, has a small respondent burden, which is particularly important for adolescent population. We recommend FFQ-m to be interview-administered, since adolescents are prone to overestimating the frequency and portion sizes of the consumed foods. Nevertheless, FFQ-m could further be adapted and validated for younger children or adults.
